# Anatomy education for medical students in the United Kingdom and Republic of Ireland in 2019: A 20‐year follow‐up

**DOI:** 10.1002/ase.2126

**Published:** 2021-12-01

**Authors:** Claire F. Smith, Samuel K. Freeman, David Heylings, Gabrielle M. Finn, D. Ceri Davies

**Affiliations:** ^1^ Department of Medical Education Brighton and Sussex Medical School University of Sussex Brighton UK; ^2^ Department of Pediatrics Royal Alexandra Children's Hospital Brighton UK; ^3^ Department of Medical Education Norwich Medical School University of East Anglia Norwich UK; ^4^ School of Medical Sciences Faculty of Biology, Medicine and Health University of Manchester Manchester UK; ^5^ Human Anatomy Unit Department of Surgery and Cancer Imperial College London London UK

**Keywords:** anatomy curricula, anatomy teaching, body donations, dissection, gross anatomy education, medical education, medical students

## Abstract

Anatomical education in the United Kingdom (UK) and Ireland has long been under scrutiny, especially since the reforms triggered in 1993 by the General Medical Council's “*Tomorrow's Doctors.*” The aim of the current study was to investigate the state of medical student anatomy education in the UK and Ireland in 2019. In all, 39 medical schools completed the survey (100% response rate) and trained 10,093 medical students per year cohort. The teachers comprised 760 individuals, of these 143 were employed on full‐time teaching contracts and 103 were employed on education and research contracts. Since a previous survey in 1999, the number of part‐time staff has increased by 300%, including a significant increase in the number of anatomy demonstrators. In 2019, anatomy was predominantly taught to medical students in either a system‐based or hybrid curriculum. In all, 34 medical schools (87%) used human cadavers to teach anatomy, with a total of 1,363 donors being used per annum. Gross anatomy teaching was integrated with medical imaging in 95% of medical schools, embryology in 81%, living anatomy in 78%, neuroanatomy in 73%, and histology in 68.3%. Throughout their five years of study, medical students are allocated on average 85 h of taught time for gross anatomy, 24 h for neuroanatomy, 24 h for histology, 11 h for living anatomy, and 10 for embryology. In the past 20 years, there has been an average loss of 39 h dedicated to gross anatomy teaching and a reduction in time dedicated to all other anatomy sub‐disciplines.

## INTRODUCTION

Anatomy has been described as the cornerstone of good medical practice (Davis et al., 2014) and the foundation for clinical studies (Sugand et al., 2010). In the 1990s, undergraduate anatomy education experienced a reduction in teaching hours and resources, when “newer” subjects, for example, molecular genetics, were introduced into the curriculum. At the same time, there was an increased focus on training in non‐technical skills such as situational awareness, teamwork and communication, decision‐making and prioritization, self‐awareness, and escalating care (Hamilton et al., 2019). As a result, there was a need to make anatomy courses more concise and reduce what was perceived as unnecessary detail (Royal Australian College of Surgeons, 2004; Turney, 2007; Davis et al., 2014; Smith et al., 2016). In 1993, the United Kingdom (UK) General Medical Council (GMC, 1993) produced the “*Tomorrow's Doctors*” document (GMC, 1993; subsequently updated as Outcomes for Graduates GMC, 2016) that set out to address medical curricula overcrowding and recommended a reduction in factual content. At a similar time in the United States (US), the Carnegie foundation recommended medical curriculum change, to increase integration of the various disciplines and to standardize learning outcomes (Irby et al., 2010; McBride & Drake, 2017). The implementation of the GMC's recommendations in UK medical schools led to growing concern about their impact, resulting in clinically important lacunae in students' and recently qualified doctors' knowledge of anatomy (Ger, 1996; Collins et al., 1994; Dangerfield et al., 2000). The presence of such “black holes” in anatomy knowledge has been described as anatomy deficit disorder (Reidenberg & Laitman, 2002) and there is evidence that it adversely affects patient safety (Goodwin, 2000; Kahan et al., 2001; Ellis, 2002; Kidder, 2002; Lynn‐Macrae et al., 2004; Older, 2004; Prince et al., 2005).

### The United Kingdom and Ireland anatomy context: Background

Medical school student numbers in the UK and Ireland are controlled by government quotas, to match the intake of students into medical schools with the requirements for newly qualified doctors in their respective health services. In response to growing healthcare needs, in 1997 the UK Medical Workforce Standing Advisory Committee recommended an increase in the number of medical students (Medical Workforce Standing Committee, 1997). As a result, in 1998, the UK government committed to the provision of 2000 additional university places to study medicine. It was recognized that there were regional needs not being met by the existing provision; therefore, a proportion of the 2000 places were allocated to new medical schools: Brighton and Sussex, Hull York, Keele, Lancaster, Norwich, Peninsula (a collaboration between Plymouth and Exeter that are now separate medical schools), Swansea, and Warwick. In a new departure for the UK, Lancaster, Swansea, and Warwick offered exclusively graduate‐entry medicine programs (meaning that they only accepted students who already held a degree, for a shortened four‐year program). Of the new medical schools, Lancaster and Peninsula decided not to use human cadavers to teach anatomy, instead relying on living anatomy (McLachlan, 2004; McLachlan et al., 2004), models, digital resources and, later, ultrasound. The new medical schools had the opportunity to base their curricula on current pedagogical thinking and favored innovations such as a problem‐based learning approach, and/or anatomy longitudinally spiraled throughout the curriculum (Evans & Watt, 2005).

The number of medical schools in Ireland remained static for a long time. However, the first new University in Ireland since 1922 was established in Limerick in 1972, and it opened a graduate entry medical school in 2007. Its medical course was established with an integrated problem‐based learning curriculum and does not use human cadavers to teach anatomy.

Against the backdrop of the changes resulting from “*Tomorrow's Doctors*” (GMC, 1993) and new medical schools opening, in 1999 Heylings (Heylings, 2002) conducted a survey to review the impact of *Tomorrow's Doctors* on anatomy education in the UK and Ireland providing a baseline before the expansion in new medical schools. The key findings of this study based on responses from 21 medical schools (75% of medical schools in the UK and Ireland at that time) were that 12 (57% of respondents) used a system‐based, four (19%) a problem‐based, and five (24%) a regional (traditional) approach to teaching anatomy. Dissection was the main teaching tool in 76% of courses, with an average of 2 h of practical teaching for every hour of lectures (Heylings, 2002). Every medical school used human cadavers to teach anatomy.

### Anatomy faculty

In addition to anatomists (for the purpose of this study defined as academics with a background in anatomy, or clinicians no longer practicing) traditionally engaged in teaching, research and scholarship (with different percentages allocated based on their job plan), some medical schools in the UK and Ireland have short‐term contract posts (typically 9–12 months) referred to as demonstratorships. These demonstratorships have traditionally been filled by recently qualified doctors, whose main role is to assist in practical classes. Such posts are often undertaken after the first two years of foundation (pre‐registration) training (F1 and F2), in what has become known informally as an “F3 year” before they begin training for a specific specialty. Nationally, there has been a rise in “F3” posts as recently qualified doctors take a year out of training (Hateley, 2016; Walker, 2020), but some demonstrators teach anatomy as part of their early specialist surgical training (Smith et al., 2018). In addition to teaching anatomy, medically qualified demonstrators provide students with the benefit of personal contact with someone who has recently qualified in medicine, and is relatively new to the clinical setting (Hanna & Tang, 2005; Smith et al., 2017a). The number of demonstrator posts declined due to their cost to universities and changes to the Royal Colleges of Surgeons Membership examinations, despite the educational value of such posts being supported by students, staff, and representative bodies (Lockwood & Roberts, 2007; White et al., 2007).

### Teaching hours

A key focus of the data gathered in surveys of anatomy education has been the number of hours dedicated to anatomy teaching. In 1989, the Anatomical Society of Great Britain and Ireland (now the Anatomical Society) undertook a review of medical undergraduate anatomy education and found that the total anatomy teaching time ranged from 309 to 371 h. A recommendation of the review was that 309 h were needed to teach anatomy, comprising 192 h of dissection if the whole body was dissected and 155 h if selective dissection was undertaken (Fitzgerald, 1992). The results of a subsequent survey conducted in 1999 (Heylings, 2002) revealed that the average number of contact hours had declined to 160 for traditional courses that undertook full body dissection with a regional approach, and 116 h for system‐based courses with selective dissection. More recently, Leveritt et al. (2016) presented data from one UK university (Nottingham), revealing that their undergraduate entry medical course comprised 98 contact hours for anatomy while their graduate entry course comprised 109.5 h, highlighting a difference within a single institution in the teaching time considered necessary to teach anatomy, and that a further reduction in anatomy teaching time nationally may have occurred. A similar trend has been reported after curriculum reform in Portugal in 2013, with a reduction in anatomy teaching hours from 309 to 180.5 (Guimarães et al., 2017). Data from other countries reveal a similar story; Australia, Canada, South Africa, and the US have all experienced a reduction in the number of hours dedicated to teaching gross anatomy and its sub‐disciplines over recent years (Kramer et al., 2008; Craig et al., 2010; McBride & Drake, 2017; Rockarts et al., 2020).

### Core syllabi

In response to the reduction in the number of hours available for teaching anatomy, it became increasingly important to define the minimum anatomy knowledge needed by new medical graduates. The American Association of Clinical Anatomists published a curriculum for the medical students of the 21st century (Leonard et al., 1996). Subsequently, in response to local requirements, the Anatomical Society of Great Britain and Ireland placed on its website its first “Core Regional Anatomy Syllabus” for undergraduate medical students in 2003. This syllabus was revised and published (McHanwell et al., 2007) and subsequently refined (Smith et al., 2016a) after being the first such syllabus to be validated by a “Delphi” process (Smith et al., 2016b) and has been acknowledged and endorsed by the UK GMC (GMC, 2016). Across the globe, a number of other core syllabi in gross anatomy, head and neck, embryology, and neuroanatomy have been published covering anatomy for medicine and allied healthcare professions (Leonard et al., 1996; Griffioen et al., 1999; Moxham et al., 2014; Tubbs et al., 2014; Moxham et al., 2015; Tubbs & Paulk, 2015; Fakoya et al., 2017; Connolly et al., 2018; Finn et al., 2018; Moxham et al., 2018; Holland et al., 2019;). Therefore, the question of what content newly qualified professionals need to know has to a large extent been addressed. However, the questions of how it is best to teach/learn anatomy and how much teaching time is needed to achieve the appropriate learning outcomes remain a matter of debate.

In view of the fact that it is now 20 years since the last major survey of anatomy teaching in the UK and Ireland (Heylings, 2002) and that informal discussions at conferences had indicated a shifting anatomy education landscape that was as yet unquantified, the aim of the current study was to determine (1) how anatomy is currently being taught to medical students in the UK and Ireland, (2) how this has changed over the past 20 years, and (3) how the teaching of anatomy varies between individual medical schools in the UK and Ireland.

### Hypothesis

In view of the reduction in anatomy teaching time between 1989 (Fitzgerald, 1992) and 1999 (Heylings, 2002), it was hypothesized that in 2019 there would have been a further reduction in the provision of anatomy teaching for medical students in the UK and Ireland in terms of lecture hours, practical hours, and staff numbers. The authors also hypothesized that the use of Technology‐Enhanced Learning (TEL) would have increased, as would the use of medical imaging techniques such as ultrasound.

## MATERIALS AND METHODS

The questionnaire employed by Heylings (2002) was used as the starting point for the current survey and further questions were then developed based on trends in anatomy teaching observed by the authors in the past 20 years and relevant literature. A draft survey was pilot tested by one university and refinements were made in light of its responses. The final survey (Supporting Information) comprised 51 questions. The survey was hosted on the University of Sussex Qualtrics XM survey software platform (Qualtrics Labs Inc., Provo, UT). Ethical approval for this study was granted by Brighton and Sussex Medical School Research Governance Ethics Committee (ER/BSMS3867/8).

A draft list of individuals responsible for the teaching of anatomy in UK and Irish medical schools was drawn up by the authors. This list was cross‐checked against lists of designated individuals (a designated individual is a person who has the legal responsibility under the Human Tissue Act in England, Wales, and Northern Ireland, to ensure that the statutory and regulatory requirements are met) for anatomical examination in England, Wales, and Northern Ireland and lists of licensed teachers of anatomy in Scotland and Ireland and amended where necessary, to ensure that the survey would be sent to the most appropriate person in each medical school (HTA, 2021).

Since 2014, eight new medical schools have been created in England (Medical Schools Council, 2018). Two of these (The University of Central Lancashire, founded in 2014 and the University of Buckingham in 2015) were founded as private medical schools with students paying approximately $47,000 USD per year, with a combined student intake total of 280 in 2019. In 2017, the University of Central Lancashire made a small proportion of its places available with bursaries funded by local partnerships. The remaining six medical schools (Aston Medical School founded in 2014 with their first intake of students in 2018, Anglia Ruskin Medical School founded in 2017 with their first intake of students in 2019, and the University of Lincoln Medical School founded in 2018 with their first intake of students in 2019. Edge Hill University, University of Sunderland, and Kent and Medway Medical School were all founded in 2019 and had their first intake of students in September 2020) are all public universities and students pay the same tuition fees as other medical schools in the UK, approximately $11,000 USD per year. At the time of the survey, all of these new medical schools were partnered with and using curricula from established medical schools, which allowed them to accept students earlier than if they developed their own curricula. In addition, some had not yet accepted students at the time of the survey. Therefore, these eight new medical schools were not included in the current study because they could either not provide a complete data set, or would have duplicated data from the “parent” medical school.

In January 2019, an invitation to contribute to the study and a link to the online survey was emailed to the lead individual identified at 39 medical schools offering medical degrees in the UK (33) and Ireland (6). The email also included a downloadable version of the survey that could be completed offline. Two reminders were sent if necessary, one in February and one in April 2019. Data were extracted from Qualtrics into Microsoft Excel^®^ (Microsoft Corp., Redmond, WA) in May 2019 and were analyzed using IBM SPSS statistical software, version 25.0 (IBM Corp., Armonk, NY). Data were tested for normal distribution and then descriptive analysis was undertaken for each question. Thematic analysis (Braun & Clarke, 2006, 2012) was used to investigate free text comments. This analysis was undertaken by one researcher (C.S.), using free node coding for each response. The free nodes were then grouped into sub‐codes to provide key themes in a categorical tree structure. The themes were then agreed with another researcher (S.F.).

## RESULTS

A 100% response rate from the 39 medical schools invited to participate was achieved. The response percentages given below are the percentages of those medical schools responding to each individual question. The response rate per question varied between 35 and 39 medical schools. Investigation of the internal consistency of the survey resulted in a Cronbach's Alpha score of 0.7.

### Anatomy faculty

In 26 (67%) medical schools, anatomy teachers were part of a larger grouping, typically either a Faculty of Medicine or Life Sciences. In one medical school, anatomy teachers were part of a Professional Studies group. The total staff pool of anatomy teachers in the UK and Ireland was 760, comprising 143 full‐time anatomists employed on education focused (teaching and scholarship) contracts (Mean = 4.6, SEM (standard error of mean) = 0.9, Min–Max = 0–23), 103 full‐time staff on mixed teaching, scholarship, and research‐focused contracts (Mean = 3.2, SEM = 0.7, Min–Max = 0–16).

**FIGURE 1:**
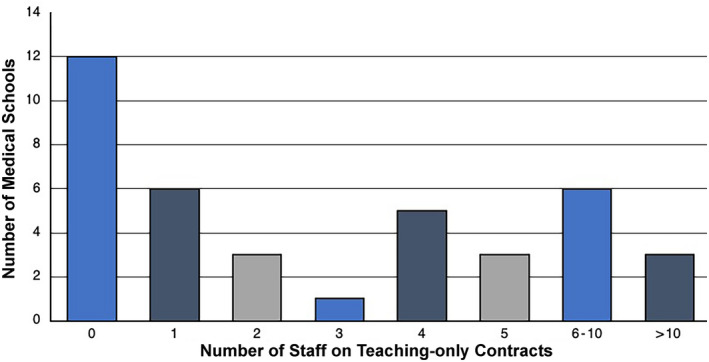
Number of staff employed on education focused contracts. This figure shows the number of staff on teaching‐only contracts (*n* = 143) employed at medical schools throughout the UK and Ireland. Staff on contracts combining teaching and research or teaching and scholarship were not included. Part‐time and full‐time employees were both counted as a single staff member (full‐time equivalents were *not* used). Where half numbers were given by institutions (due to staff being employed part‐time), numbers were rounded up to the nearest integer

The total number of anatomy demonstrators in the UK and Ireland was 407; 118 were full‐time and comprised 98 medically qualified (Mean = 2.6, SEM = 0.7, Min–Max = 0–21) and 20 non‐medically qualified (Mean = 0.5, SEM = 0.3, Min–Max = 0–10). There were 289 part‐time demonstrators, comprising 216 medically qualified (Mean = 5.54, SEM = 1.9, Min–Max = 0–50) and 73 non‐medically qualified (Mean = 1.8, SEM = 0.9, Min–Max = 0–30). In addition, there were 107 other part‐time anatomy teaching staff (Figure 1).

### Curriculum and contact hours

**FIGURE 2:**
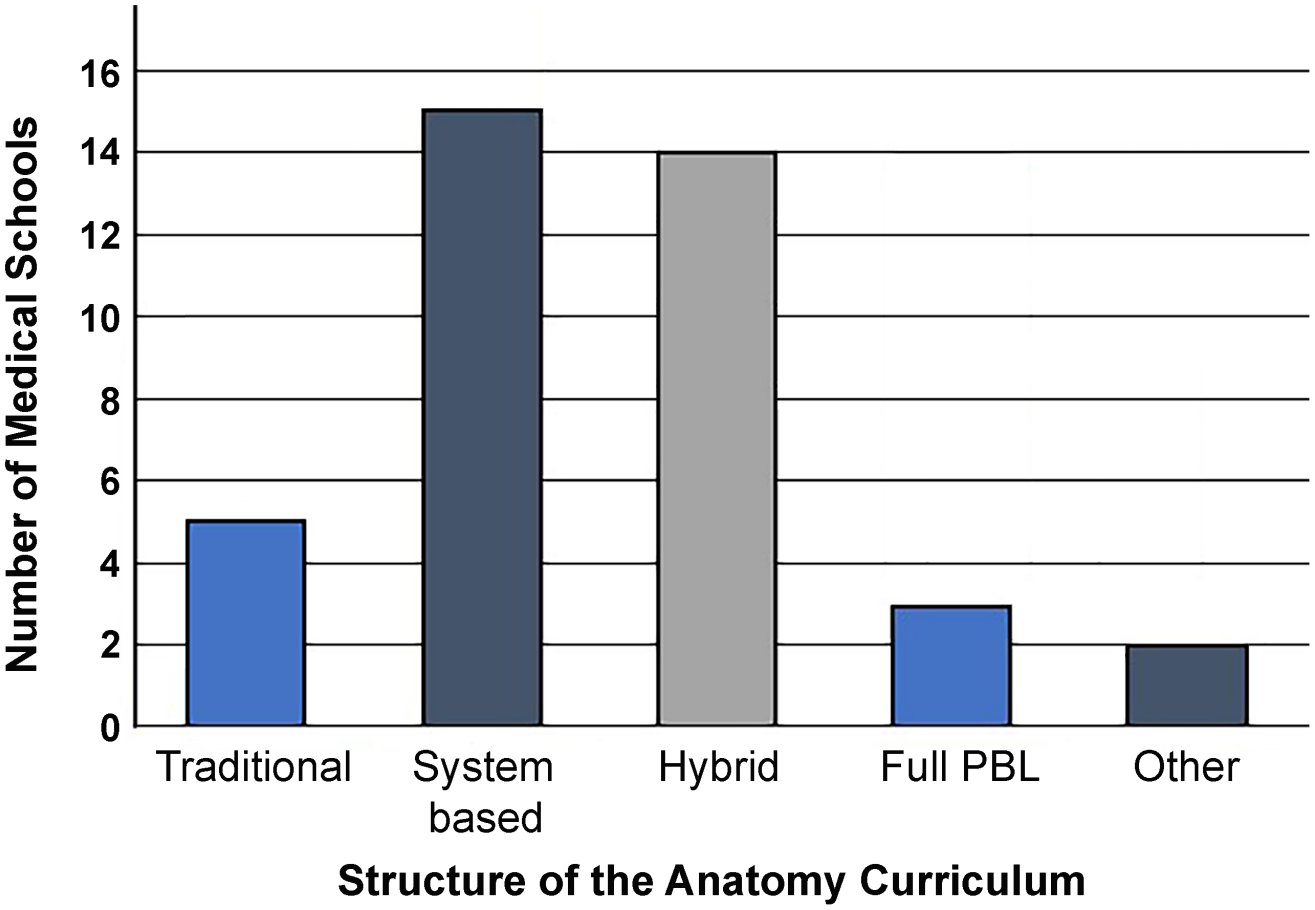
The proportion of medical schools (*n* = 39) using various teaching methods to structure their anatomy curriculum. Traditional refers to a regional‐based approach. The predominant approaches were systems based and hybrid. PBL, problem‐based learning

The number of medical students enrolled in each medical school varied from 71 to 450 per annum. The annual intake for each medical school is given in Table 1. In all, 20 (51%) medical schools provided the option of an intercalated/integrated bachelor degree and in seven medical schools the BSc was a compulsory part of the medical course. The type of anatomy curriculum was categorized as regional/traditional based, system‐based, full problem‐based learning, or hybrid. A hybrid curriculum was defined as one that combined components of the other types in any proportion. The curriculum categories used in the current study followed those of Heylings (2002) and remain a helpful overall indicator of the type of curriculum employed. The majority of medical schools employed either system‐based or hybrid curricula, with just five medical schools delivering a regional curriculum (Figure 2). Free comment responses provided information about hybrid curricula, for example, “regional anatomy in a system‐based course” and “lectures are system‐based and practicals are regional.” When asked about the level of input into and autonomy over the curriculum, 4 (10%) respondents indicated they did not have any control over the curriculum, 23 (59%) reported not having control over the approach to teaching used, and 14 (36%) did not have any control over the teaching time allocated to anatomy. All respondents indicated that anatomists were responsible for the gross anatomy content of the curriculum, compared to 56% reporting anatomists having control over histology, 76% over embryology, 83% over neuroanatomy, 78% over living anatomy, and 56% over medical imaging content.

**TABLE 1:** Participating medical schools and medical student cohort numbers

Institution	Number of medical students per cohort	Number of donors per annum
Brighton and Sussex Medical School	200	30
Cardiff University	315	40
University College Cork	205	27
Hull York Medical School	225	30
Imperial College London	350	16
Keele University	150	25
Kings College London	450	80
Lancaster University	71	0
National University of Ireland Galway	196	20
Newcastle University	375	30
Norwich Medical School	208	11
Plymouth University	160	0
Queen Mary University of London	370	53
Queen's University Belfast	270	30
Royal College of Surgeons Ireland	420	27
St George's University of London	255	55
Swansea University	100	0
The University of Edinburgh	250	45
Trinity College Dublin	196	13
University College Dublin	300	20
University College London	334	20
University of Aberdeen	183	42
University of Birmingham	400	20
University of Bristol	270	100
University of Cambridge	320	55
University of Dundee	210	90
University of Exeter	148	0
University of Glasgow	300	100
University of Leeds	270	70
University of Leicester	270	50
University of Limerick	150	13
University of Liverpool	255	35
University of Manchester	420	36
University of Nottingham	380	55
University of Oxford	185	32
University of Sheffield	254	60
University of Southampton	260	17
University of St Andrews	225	16
University of Warwick	193	0
Total	10,093	1,363

The main reported constraints on anatomy teaching were coded into the following two themes: (1) anatomy was not a standalone component and had to fit in with the whole curriculum and (2) timetabling constraints determined when anatomy could be taught. These both reflect practical limitations. However, the fact that anatomy was not a standalone component may have been a positive development, reflecting subject integration. Only two (5%) medical schools reported that anatomy was a standalone component of their curricula. Six (14%) respondents stated that it was difficult to identify clear anatomical components within their curricula. These respondents were predominantly from medical schools employing problem‐based learning. Gross anatomy teaching was integrated with medical imaging in 39 (95%), embryology in 33 (81%), living anatomy in 32 (78%), neuroanatomy in 30 (73%), and histology in 28 (68%) medical schools.

In total, 11 medical schools were unable to identify how many contact hours are dedicated to teaching gross anatomy because of the nature of their curricula. The contact hours for the remainder ranged from 30 to 145 h (Mean = 85.3, SEM = 5.9). For histology, they ranged from 2 to 104 h (Mean = 23.6, SEM = 5.4); for embryology from 1 to 20 h (Mean = 9.5, SEM = 1.4); for neuroanatomy 6 to 71 h (Mean = 23.9, SEM = 3.5); and for living anatomy 2 to 65 h (Mean = 10.5, SEM = 3.2).

### Teaching method and assessment

Topographical (gross) anatomy was predominantly taught in Years 1 and 2 of the medical curriculum (58%), with 14 medical schools (37%) teaching gross anatomy over a longer time period (Table 2). The predominant practical teaching approach was dissection in one (2%) medical school, prosection in nine (22%), a combination of dissection and prosection in 14 (34%), TEL‐based methods in three (7%) and anatomical models in four (10%). Eight (20%) medical schools predominantly either used other approaches (including pathology and anatomical pots, ultrasound, living anatomy) or they could not identify a predominant approach. Two (5%) medical schools did not provide information on their approach to practical teaching.

**TABLE 2:** The distribution of anatomy and its sub‐disciplines within the curriculum at UK and Irish Institutions

Discipline	Year 1 Only *n* (%)	Year 1 and 2 *n* (%)	Year 1, 2 and beyond *n* (%)
Gross Anatomy	2 (5)	22 (58)	14 (37)
Histology	8 (22)	23 (64)	5 (14)
Embryology	17 (46)	15 (41)	5 (14)
Neuroanatomy	6 (16)	21 (55)	11 (29)
Living Anatomy	7 (21)	14 (41)	13 (38)

**FIGURE 3:**
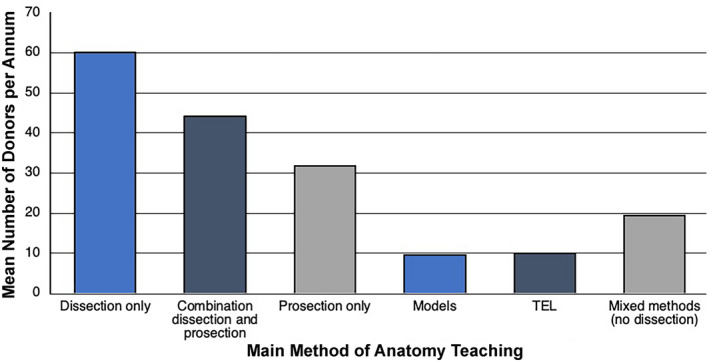
The mean number of cadavers donated for each predominant method of teaching anatomy to medical students. Dissection uses the highest number of cadavers. TEL, technology‐enhanced learning

In all, 34 (87%) medical schools reported that they used human cadavers for teaching, with a requirement of 1363 bodies per annum. In all, 32 of the 34 (94%) medical schools that used cadavers employed formalin as the primary fixative and eight used one of a variety of soft embalming techniques. An overlap between the use of specimens for undergraduate and postgraduate courses was frequently reported. It is perhaps not surprising that full body dissection was the teaching method that used the greatest number of cadavers (mean = 60) per annum (Figure 3). In all, 20 (59%) medical schools had an element of dissection in their courses, with a mean of 118 students per practical class (range: 20–250) and a mean of nine members of teaching staff (range: 2–19). In these medical schools, some regions were not dissected, for example five medical schools did not dissect the head and neck or the pelvis.

**FIGURE 4:**
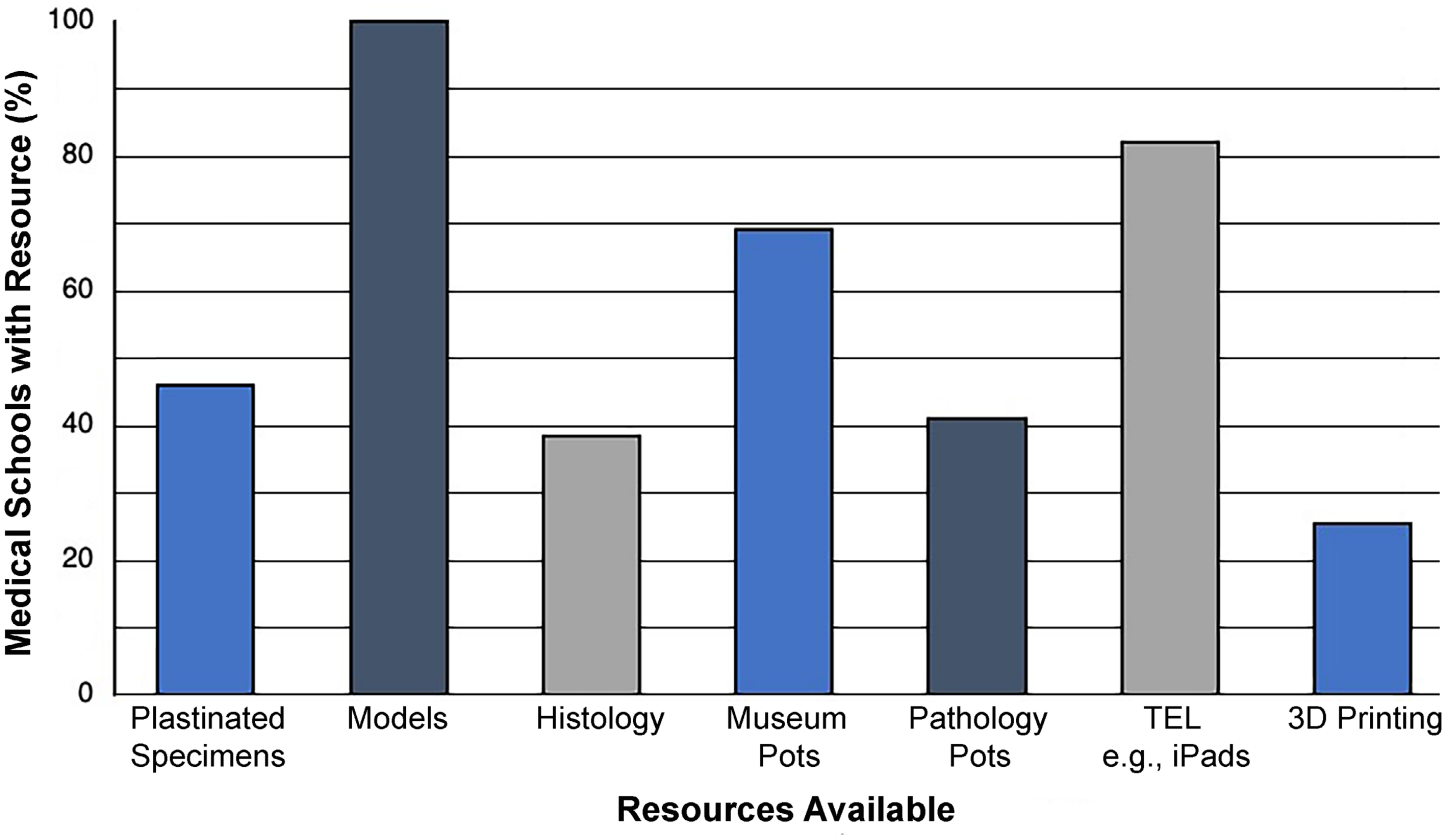
The availability of various resources during anatomy classes for medical students. Histology refers to both physical histology slides and virtual microscopy images. All medical schools use models and a significant amount now use iPad/tablets. Number of responses (*n* = 39). TEL, technology‐enhanced learning; 3D, three‐dimensional

Dissection classes were frequently repeated to accommodate all students, with up to six repetitions at one medical school. Medical schools that used prosection as the principal means of practical anatomy teaching (*n* = 20) had a mean of 69 students per class (range: 20–150), who were supported by an average of six teaching staff (range: 1–25). Prosection classes were repeated up to 10 times at one medical school. Due to the fact that donor cadavers were also used by other allied healthcare courses and usage between courses differed within institutions, it was not possible to determine student:donor cadaver ratios. All medical schools had anatomical models available to aid the study of anatomy and 82% had some form of TEL, for example, iPads (Figure 4). Twenty five percent supported learning with three‐dimensional (3D) printing and 16% supported learning with a museum area.

In total, 36 medical schools reported that they had a form of summative assessment of anatomical knowledge, with 28 (77%) using assessment at the end of a trimester/semester/module/unit. The most common method of assessment was a multiple choice question (MCQ) paper, used in 33 (84%) of medical schools. The term MCQ has been used here to cover a variety of question types that do not involve free text answers, including single best answer, true or false, and extended matching questions. Anatomy spot tests were used in 19 (51%) medical schools. Progression was based on performance in a standalone anatomy examination in only 7 (18%) of medical schools. In 25 (69%) medical schools, integrated assessments were employed that allowed students to pass and progress even if they failed the anatomy component. In 37 (97%) medical schools, anatomists designed the questions used in assessments, in 36 (95%), anatomists reviewed questions set by others and in 28 (74%), anatomists were involved in the marking process. Anatomists submitted questions to be used in Objective Structured Clinical Examinations in just over half (21 [55%]) of the medical schools surveyed. In only 14 (36%) medical schools was there any summative assessment of anatomical knowledge after the first two years of the medical course.

All medical schools offered a form of formative assessment, predominantly at the end of a trimester/semester/module/unit. MCQ were the most popular type of formative assessment (25 medical schools, 68%) and 21 (60%) offered a formative spot test. Other types of formative assessment included written questions, case‐based discussions, and viva voce assessments. In addition to the end of a trimester/semester/module/unit formative assessments, regular digital spot tests, online testing, and mini spot tests in individual practical classes were also employed.

The penultimate question of the survey asked “In recent years what areas of anatomy teaching has your institution invested in e.g., TEL, soft embalming etc.?” Thematic analysis highlighted three principal areas: TEL, physical laboratory infrastructure, and ultrasound. Responses particularly focused on improvement of the learning environment, and resource procurement and utilization “we have improved the environment in the DR (dissecting room, i.e., anatomy laboratory) with new equipment and are introducing tablets primarily to be used by the demonstrators and academics leading the session.” Responses to the final question, “What is your biggest concern for the future of anatomy?” highlighted three principal concerns: (1) reduced teaching time in the curriculum, (2) the cost of using cadavers, and (3) the lack of availability of suitably qualified staff. Comments such as “Pressure to ditch full body dissection and move on to virtual reality” and “Loss of dissection facilities due to financial pressures” and “Premature termination of body donation programs before satisfactory technological replacements have been perfected” reflect the major concerns of anatomists.

## DISCUSSION

### Anatomy faculty

In the context of the current study, an anatomist can best be defined as an academic engaged in the teaching of anatomy. The literature reflects concern over the difficulty in recruiting anatomists (Cahill & Leonard, 1999; Dyer & Thorndike, 2000), with some anatomy departments resorting to hiring teaching staff without training or experience (Cottam, 1999). Anatomists have also retired and have not been replaced (Dyer & Thorndike, 2000), resulting in a “greying anatomy faculty” (Topp, 2004). Programs like the Anatomy Training Program set up between the Anatomical Society (UK and Ireland) and the American Association for Anatomy have aimed to provide training in anatomy to junior scientists. Yet to this day, there remains a shortage of anatomy teachers (Wilson et al., 2020). In agreement with the results of the 1999 survey (Heylings, 2002), those of the current study revealed that large differences in staff numbers remained between medical schools. However, the results of the current study also revealed an overall reduction in the total number of anatomy teachers in UK and Irish medical schools over the past 20 years, despite the fact that there has been a considerable increase in the number of new medical schools and medical students since 1999. When comparing all anatomy teaching faculty (full‐time and part‐time, excluding demonstrators), the staff pool decreased from an average of 11 per medical school in 1999 to 9 in 2019. These numbers are similar to the average of 11 staff per medical school reported for Australia (Craig et al., 2010). The results of the current study revealed that demonstrator numbers have increased since 1999 and included 29 non‐medically qualified anatomy demonstrators, suggesting a possible shift to less reliance on medically qualified demonstrators.

### Curriculum and contact hours

Until relatively recently, three main types of curricula have existed: regional, systems‐based, and problem‐based. These three types of curricula have been typically associated with different methods of teaching anatomy. For example, a regional curriculum (sometimes referred to as a traditional curriculum) has been associated with dissection (Nnodim, 1997). Over the past 20 years in the UK and Ireland, there has been a shift toward systems‐based and hybrid curricula.

The trend toward medical courses moving to systems‐based curricula, as promoted by “*Tomorrow's Doctors*” has been criticized for resulting in a decrease in attainment (McKeown et al., 2003). Comparison of the effectiveness of integrated and standalone curricula approaches in the US using performance in the United States Medical Licensing Examination^®^ (USMLE^®^), revealed that students who studied using an integrated curriculum performed significantly worse than those who studied a standalone regional/traditional curriculum (Cuddy et al., 2013). The regional approach has stood the test of time, but integrated curricula have aimed to integrate not only the curriculum content but also assessment and to encourage the interaction between students, core faculty, and clinicians (Reidenberg & Laitman, 2002). This survey clearly demonstrates the effect of integration with 69% stating that students are able to progress without achieving a pass in anatomy questions. Therefore, it is perhaps not surprising that Estai and Bunt (2016) considered that the best way to teach anatomy is to combine multiple approaches, giving rise to the “hybrid” curriculum.

Anatomy has traditionally been studied in the early years of the medical curriculum and in 1999 gross anatomy was predominantly (57%) taught over the first two academic years, with 36% of universities teaching anatomy in one year and only one medical school teaching anatomy for a period of greater than two years. In 2019, 22 (58%) medical schools taught anatomy over two years and only two (5%) over one year. In all, 14 (37%) medical schools taught anatomy for longer than two years. In a study of 13 UK medical schools in 2005, Gogalniceanu et al. (2009) reported that only two taught anatomy beyond year two and the results of the current study reveal that by 2019, 33% did. This shift is likely a result of attempts to introduce clinical skills and patient contact earlier in curricula, with anatomy being spread over a longer time period to accommodate this change. It may also be a consequence of spiral and more integrated curricula being adopted.

The debate about the number of contact hours required to teach anatomy to ensure graduates are safe and competent practitioners is ongoing. The results of the current study of UK and Irish medical schools revealed that between 1999 and 2019 there was a mean loss of 39.18 h of anatomy teaching time from the curriculum. A similar trend has occurred in the US, with a reduction of 38 h of anatomy teaching time between 1997 and 2017 (McBride & Drake, 2017), possibly in response to *“Accelerating Change in Medical Education”* (AMA, 2015). However, although the reduction in gross anatomy teaching time is similar in the UK and Ireland compared to the US, the total number of teaching hours are very different, with a mean of 129 h in the US in 2017 (McBride & Drake, 2017), compared to 85 h in the UK and Ireland in 2019. Furthermore, the literature demonstrates that Canada had 9% more gross anatomy teaching hours (Rockarts et al., 2020) and Craig et al. (2010) reported and Australia and New Zealand had double the anatomy teaching hours compared to the UK and Ireland.

The results of the current study revealed that the teaching hours for histology varied greatly (2–104 h) between UK and Irish medical schools, but the mean of 23.6 h is under half the average of 51 h reported for the US (McBride & Drake, 2017), but quite similar to the 25.2 h in Canada (Rockarts et al., 2020). The current results revealed a marked difference in neuroanatomy teaching hours, with the US spending over 200% more time (80 h) (McBride & Drake, 2017) than the UK and Ireland. The teaching hours for embryology in the UK and Ireland in 2019 were similar to those in the US (McBride and Drake, 2017), averaging 10 and 14 h, respectively, but more than the 7.4 h in Canada (Rockarts et al., 2020).

The results of the current survey highlighted the large variation between medical schools in the number of contact hours for teaching gross anatomy (30–145 h [Mean = 85.3, SEM = 5.9]) and its sub‐disciplines, reflecting considerable diversity in approaches to teaching anatomy in the UK and Ireland. In the UK, the national Medical Licensing Assessment (MLA) will be implemented in 2023–2024 and students must pass this to enter Foundation Training after graduation. There has been some criticism that the MLA will stifle the ability of universities to produce doctors with differing strengths and that it might create an assessment lead minimum curriculum that each medical school will focus on. However, the counter argument is that the MLA will produce doctors with the same minimum standards (McCrorie & Boursicot, 2009), by helping to detect poorly performing students (Devine et al., 2015) and thus, drive up standards (Hateley, 2015). National Licensing Examinations are commonplace in many countries (Archer et al., 2017), including the USMLE^®^ in the US. Archer et al. (2017) suggested that such examinations should focus on a balance between assessing breadth of skills and the capacity to use these skills in practice. The same focus can be applied to anatomy; with such a breadth of content to cover, it is almost impossible to list everything that will ever be needed in practice; therefore, the focus should be on application in practice. However, students and newly qualified doctors consider anatomical knowledge important. In Australia, the country that has the highest anatomy teaching hours globally, the Australian Medical Student Association reported that 73% of students thought that anatomy teaching hours were too small, and only 40% of students reported that they would graduate with sufficient knowledge of anatomy (Craig et al., 2010). Similar views were echoed in the UK when nearly qualified and just qualified doctors were asked to estimate how much of the Anatomical Society's Core Regional Anatomy Syllabus (Smith et al., 2016) they knew; only 46% reported that they knew over 50% of its learning outcomes (Smith et al., 2019).

The decreasing number of hours allotted to anatomy education as a medical student has created an increasing focus on anatomy at postgraduate levels. For example, the introduction of the London Postgraduate School of Surgery's compulsory Core Surgical Anatomy course for all surgical trainees in the first year of their core training and its requirement for all year 2 core trainees to spend time demonstrating anatomy to medical students (Smith et al., 2018). These requirements were reinforced by an 8.3% increase in the anatomy content of the Part A (written examination) of the Intercollegiate Membership Examination of the Royal Colleges of Surgeons in 2017 (Brennan & Smith, 2016), because of concern that the amount of anatomical knowledge required to pass the examination was an insufficient basis for postgraduate surgical training (Smith & Brennan, 2013).

### Teaching methods and assessment

#### Lectures

Lectures have historically been regarded as a principal method of delivery of medical education. With the reduction in teaching contact hours described above, it is not surprising that the number of gross anatomy lectures has also decreased over the past 20 years in the UK and Ireland, from an average of 70 in 1999 to 53.6 in 2019. For region‐based courses, this trend is mirrored for the sub‐disciplines of anatomy. However, there has been an increase in the number of histology, embryology, and neuroanatomy lectures for systems‐based courses (histology increased from 11.2 to 14.7, embryology from 5.7 to 8.6, and neuroanatomy from 9.2 to 11.9), possibly reflecting the need to address material that had been previously removed. Some medical schools have used team‐based learning to replace lectures and to increase the interaction in teaching sessions (Vasan et al., 2008). More recently, video conferencing, for example, via Google Hangouts (Moszkowicz et al., 2020), has been employed to deliver lecture style sessions and the response to the recent global Covid‐19 pandemic has resulted in anatomy lectures being delivered through video platforms such as ‘Panopto’ (Panopto Inc., Seattle, WA) and “Zoom” (Zoom Voice Communications Inc., San Jose, CA; Longhurst et al., 2020).

#### Use of human cadavers

The usefulness of human cadavers for learning anatomy has been a matter of considerable debate (Aziz et al., 2002; Granger, 2004; McLachlan, 2004; Patel & Moxham, 2006; Fitzgerald et al., 2008; McMenamin et al., 2018). Meta‐analysis has shown that in terms of assessment outcomes, it is not superior to other methods of learning anatomy (Wilson et al., 2017). However, some authors have stated its importance as a “rite of passage” (Dyer & Thorndike, 2000) or a “royal road” (Newell, 1995) and others that it is a form of learning that imparts more than just factual knowledge (Smith et al., 2020). Moreover, dissection itself is changing as it evolves to reflect the latest clinical practice (Cotofana & Lachman, 2020) and during the Covid‐19 pandemic, many educators have had to explore new ways for students to gain experience of human cadavers, for example, with material provided for asynchronous learning, to enable a stronger focus when students are in the anatomy laboratory (Smith and Pawlina, 2021).

With the current study showing 87% of UK and Irish medical schools compared to 100% of medical schools in the US, McBride and Drake (2017) used cadavers in some form, it is not surprising that student perceptions toward cadavers in the UK have shown 60%–94% hold a positive attitude (Quince et al., 2011). Forty‐one percent of medical schools offered dissection in the UK and Ireland in 2019, a much lower figure than the 79% of schools in Australia (Craig et al., 2010) and 100% of medical schools in the US (McBride & Drake, 2017) reported using human cadavers in some form. It is interesting that there has been an increase in the use of Thiel (Thiel, 2002) or other types of “soft” embalming of cadavers for teaching medical students. It has been suggested that “soft” embalmed cadavers give a more “clinical” experience and can be used for other activities in addition to undergraduate teaching (Eisma et al., 2013; Balta et al., 2014), but there is also evidence that students find it more difficult to identify the more mobile structures in “soft” embalmed cadavers (Balta et al., 2014). In 2019, 55% of those medical schools employing dissection reported that more than one group (in a different class) worked on a cadaver, with one group dissecting superficial structures and then a second group dissecting deeper. Furthermore, 76% reported that more than one group worked on a cadaver, studying different regions. This possibly highlights the way cadavers are now being utilized to accommodate increasing numbers of students and to make the maximum use of the cadavers.

#### New teaching/learning methods

It is impossible to explore changes in learning provision in anatomy over the last 20 years without considering the rise of innovation, including both the rise of arts and humanities, for example, body painting (Finn & McLachlan, 2010), clay modeling (Oh et al., 2009; Bareither et al., 2013; Curlewis et al., 2021), and also technology. Globally, there has been an explosion in the use of TEL and the use of personal electronic devices (Swedin & Ferro, 2007). TEL was first used in anatomy education in the early 1990s, most notably in an attempt to enhance spatial understanding (Garg et al., 1999) and to create virtual microscopy sessions (Kumar et al., 2006). Videos and medical imaging can also easily be mapped to TEL applications (Trelease, 2016). The possibility that TEL could replace the traditional anatomy laboratory has been raised, but the use of computer‐generated three‐dimensional images to learn the anatomy, for example, of the ear, has been questioned (Nicholson et al., 2006) and there is concern that current computed‐generated anatomy content lacks the normal individual variation of the human body so important for clinical students to appreciate. Nevertheless, the current study revealed that in the UK and Ireland in 2019, 82% of medical schools utilized iPads or other tablets in laboratory sessions, with similar trends in the US, where 44% of medical schools used virtual or video dissections for teaching (McBride & Drake, 2017). Three‐dimensional anatomical models have also become embedded in the UK and Ireland (McMenamin et al., 2014; O'Reilly et al., 2016; Smith et al., 2017b). There has been a marked increase in the availability of 3D virtual resources for teaching/learning anatomy, including digitized cadaveric resources. There has also been an increase in learning anatomy from global social media in the form of anatomy video clips on YouTube (Barry et al., 2016), and the use of Facebook (Jaffar, 2014) and Twitter (Hennessy et al., 2016) to promote engagement.

#### Assessment

Assessment in clinical education has clear implications for patient safety. Along with the desire to increase integration in curricula came a move to integrate subject assessment. As a result, there has been a reduction in the number of medical schools using identifiable anatomy questions in regional courses in the UK and Ireland from 75% in 1999 to 25% in 2019 and in system‐based courses from 42% in 1999 to 33% in 2019. In both 1999 and 2019, none of the courses described as being problem‐based learning used identifiable anatomy questions. There has been a notable increase in medical schools using MCQ type assessments, from a mean of 60% for region‐based courses in 1999 to 80% in 2019 and from 75% to 81.2% for system‐based courses; this may reflect a drive to increased standardization and automation of the marking process and/or the reduction in the number of staff available to mark free text or essay style answers. This usage is similar to that in Canada, where 75% of universities used MCQs in anatomy assessment (Rockarts et al., 2020), and in Australia and New Zealand where 84% of universities used MCQs (Craig et al., 2010).

An assessment typically used to examine anatomy knowledge is the practical examination referred to variously as a “spotter,” “pin and flag,” or “steeplechase.” It is interesting to note that the use of this type of practical examination increased from 63% of all medical school courses in the UK and Ireland in 1999 to 74% in 2019. This use of anatomy practical examinations is greater than that reported for Australia and New Zealand (47% of universities; Craig et al., 2010). The increase in the use of spotter style examinations in the UK has previously been reported in the literature (Smith & McManus, 2014; Sagoo et al., 2016) and the use of images in online spotter style assessment improves students' performance (Sagoo et al., 2021). Using online images, anatomy can also be integrated with assessment of other disciplines and clinical content (Yaqinuddin et al., 2012).

### Class size

With increasing medical student numbers, medical schools are faced with the problem of how to accommodate more students and still retain a “personal” and high‐quality learning experience. In 1999 (Heylings, 2002), the average dissection class had 6 members of staff and 129 students (ratio 1:19.8). Twenty years later, the results of the current study reveal that the average dissection class had nine staff and 118 students (ratio 1:14). This is a slightly lower ratio than in the US (1:19, McBride & Drake, 2017). This apparent improvement in the staff student ratio in the UK and Ireland may be accounted for by the increase in the number of part‐time staff including anatomy demonstrators but it also may be a reflection of the need for more staff to counter the effects of reduced teaching time. Increased students numbers has meant that the size of the anatomy laboratory has become a limiting factor. This has resulted in an increase in the number of students per group, for example, in 1999 there was a mean of 62 students in systems‐based prosection classes, but by 2019 this had increased to 96 students. It might have been expected that the number of repetitions of practical classes would also have increased, but they had not. This possibly suggests that lessons have been learned about multiple class repetitions offering a decreasing standard of education due to teacher fatigue.

#### Limitations of the study

A limitation of the current study is that the previous survey in 1999 (Heylings, 2002) only achieved a response rate of 75%; hence, comparisons between it and the current survey do not include the “unknown 25%” of medical schools in the previous survey. Each medical school had a different number of students, hence comparing staff‐to‐student ratios would have been informative, but these data were not collected in 1999. Furthermore, in the current survey, staff‐to‐student ratios in medical schools often varied from one teaching session to another and therefore, it was not possible to report staff‐to‐student ratios accurately. The current survey was undertaken before the onset of the Covid‐19 pandemic resulted in major changes to anatomy teaching provision. These changes are unlikely to have affected anatomy course learning outcomes, but have changed the mode of delivery (Brassett et al., 2020; Longhurst et al., 2020; Pather et al., 2020; Smith & Pawlina, 2021) and the number of available donors. It remains to be seen whether when Covid‐19 restrictions are lifted, there will be a return to the type of anatomy education provision described in the current study.

## CONCLUSION

In the past 20 years, there has been a marked increase in the number of medical students and new medical schools in the UK and Ireland and over the same time there has been a shift in the type of person teaching them, with an increase in the number of part‐time staff including medical demonstrators without a proportionate increase in permanent academic staff numbers. Reduced teaching contact hours has meant that anatomists have had to adapt and be creative in how they plan and deliver teaching; the increasing use of TEL has provided some solutions to curriculum changes. The survey of Heylings (2002) and the current survey only covered the provision of pre‐qualifying medical anatomy teaching; however, it is clear that cuts in undergraduate anatomy curricula have resulted in an increased need for anatomy teaching in postgraduate curricula, particularly in surgical and radiological training. Perhaps now is the time to have conversations about what we want anatomy education to look like in 20 years' time to be best suited to the needs of our future medical students and doctors.

## Supporting information

Supplementary Material
