# Supplementary material for: Anatomy education for medical students in the United Kingdom and Republic of Ireland in 2019: A 20‐year follow‐up
Source: Anat Sci Educ. 2021 Dec 1;15(6):993–1006. doi: 10.1002/ase.2126 (PMC9786311; doi:10.1002/ase.2126)
Supplement: Supplementary file 1 — Supplementary Material [file ASE-15-993-s001.docx]

Appendix A – Survey sent to all UK and Irish university anatomy departments

1. What is the name of your institution?
2. What country is the institution located in?
3. Is anatomy a defined group/department at your institution? Y/N
4. What larger group is anatomy part of e.g. faculty/college?
5. How many staff are on teaching only contracts?
6. How many staff are on mixed teaching and research contracts?
7. For full-time teachers only, please give figures for each of the following:

- Number of gross anatomy teachers medically qualified:
- Number of gross anatomy teachers dentally qualified:
- Number of other gross anatomy teachers (with other qualifications e.g. MSc, PhD):
- Number of annually appointed medically qualified demonstrators:
- Number of annually appointed demonstrators not medically qualified:
- Number of other staff not included above:
- Total:

1. For part-time teachers only, please give figures for each of the following:

- Number of gross anatomy teachers medically qualified:
- Number of gross anatomy teachers dentally qualified:
- Number of other gross anatomy teachers (with other qualifications e.g. MSc, PhD):
- Number of annually appointed medically qualified demonstrators:
- Number of annually appointed demonstrators not medically qualified:
- Number of other staff not included above:
- Total:

1. How many students are registered to study medicine each year?
2. How many students are on an undergraduate programme?
3. How many students are on a postgraduate programme?
4. Does the medicine course include an integrated/intercalated BSc? Y/N
5. If so, is this BSc compulsory? Y/N
6. The anatomy curriculum for medical students was taught predominantly by which method last year?
   - Traditional (e.g. regional)
   - Systems-based
   - Full problem-based learning
   - Hybrid
   - Other
7. If hybrid or other, please explain.
8. On medicine courses, the anatomy staff have control of which of the following?

- The curriculum contents
- The approach used for teaching
- The teaching time allocated
- The ratio of lectures to practical sessions

1. If other constraints are imposed, please explain.
2. Is it possible to clearly identify anatomical components in the curriculum? Y/N
3. If yes to Q18, please give figures for each of the following:

- Total contact hours per student for gross anatomy:
- Total contact hours per student for histology:
- Total contact hours per student for embryology:
- Total contact hours per student for neuroanatomy:
- Total contact hours per student for living anatomy:
- Total contact hours per student:

1. If no to Q18, please state total contact hours:
2. Please explain anything further if you wish:
3. Anatomists are responsible for the delivery of which of the following:

- Gross anatomy
- Microanatomy (histology)
- Embryology
- Neuroanatomy
- Living anatomy
- Imaging
- Clinical skills

1. Are each of the following subjects taught over one academic year, two academic years or a longer period of time within the medical curriculum?

- Gross anatomy
- Microanatomy (histology)
- Embryology
- Neuroanatomy
- Living anatomy

1. For the medicine course, your gross anatomy teaching approach is predominantly based on:

- Dissection only
- Prosection only
- A combination of dissection and prosection
- Imaging
- Technology-enhanced learning (TEL)
- Models
- Other

1. If other, please explain.
2. The gross anatomy teaching is integrated with which of the following:

- Histology
- Embryology
- Neuroanatomy
- Living anatomy
- Imaging
- Other disciplines e.g. physiology, pharmacology
- Clinical skills teaching
- Nothing (a stand-alone course)
- Other

1. If other, please explain.
2. If you use human tissue, please state the type of preservation: formalin, soft, fresh frozen?
3. If you use human tissue, please state the number of donors you accept per annum.
4. If you predominantly dissect:
5. How many students are in each class?
6. How many staff (all categories) are in each class?
7. How many of these staff are demonstrators?
8. How many near-peer teachers do you have?
9. How many times is each class repeated?
10. What is the ratio of students to cadaver?
11. Does more than one group (in a different class) work on the same cadaver in the same region e.g. group A does superficial, group B does deep?
12. Does more than one group (in a different class) work on the same cadaver in a different region e.g. group A does thorax, group B does head?
13. Are the cadavers prepared before class i.e. skin flaps?
14. If you organise prosections:
    1. How many students are in each class?
    2. How many staff (all categories) are in each class?
    3. How many of these staff are demonstrators?
    4. How many near-peer teachers do you have?
    5. How many times is each class repeated?
    6. If students work in groups, how many are in each group?
15. If you use a hybrid approach, please explain more
16. Which of the following are available during anatomy classes?
17. Plastinated specimens
18. Models
19. Histology slides/virtual microscopy
20. Museum pots
21. Pathology pots
22. TEL e.g. iPads
23. 3D printing
24. Do you have identifiable anatomical lectures? Y/N
25. For each classification of lecture, please state the number of hours over the curriculum:

- Gross anatomy
- Histology
- Embryology
- Neuroanatomy

1. Do you support teaching with a museum display area? Y/N
2. Regarding teaching medical students, is there anything you wish to add about any aspect not covered?
3. How do you summatively assess your students’ anatomical knowledge?

- End of semester/module examination only
- End of anatomy course examination i.e. assessing the whole body
- Through a practical spot examination only
- Through identifiable anatomy written questions only
- Through questions in an integrated case-based paper only
- Through an MCQ/SBA/EMI paper only
- Through a viva

1. How do you formatively assess your students’ anatomical knowledge?

- End of semester/module examination only
- End of anatomy course examination i.e. assessing the whole body
- Through a practical spot examination only
- Through identifiable anatomy written questions only
- Through questions in an integrated case-based paper only
- Through an MCQ/SBA/EMI paper only
- Through a viva

1. If you use any other method of assessment, please explain.
2. Regarding curriculum assessment, students proceed to the next stage of the course by:

- Passing a specific standalone anatomy assessment
- Passing an integrated year paper when they must have an overall pass across the anatomy questions
- Passing an integrated year paper when they could have an overall fail across the anatomy questions

1. Is anatomical knowledge formally assessed later in the curriculum when formal anatomy teaching has ceased? Y/N
2. With regard to anatomy assessment:
   1. Do you design the questions yourself?
   2. Do you review questions set by others?
   3. Are your questions used in OSCEs/OSPEs?
   4. Are you asked to be involved in marking these questions?
   5. Are your questions used in a written paper?
3. Is there any anatomy teaching which is delivered in an Interprofessional Education format? Y/N

- If yes, please explain.

1. For other undergraduate anatomy sessions, please select the course, number of students, the number of hours per student, and the main method of teaching (dissection, prosection, hybrid or lecture). Leave blank if not applicable:
   - BSc Anatomy
   - BSc Art
   - BSc Biomedical Science
   - BSc Chiropractic
   - BSc Dental Sciences
   - BSc Dental Hygiene
   - BSc Midwifery
   - BSc Neuroscience
   - BSc Nursing
   - BSc Occupational Therapy
   - BSc Oral Health Sciences
   - BSc Physiotherapy
   - BSc Podiatry
   - BSc Radiography
   - Speech and Language Therapy
2. Please give details about any other undergraduate courses not listed above.
3. For other postgraduate anatomy sessions, please select the course, number of students, the number of hours per student, and the main method of teaching (dissection, prosection, hybrid or lecture). Leave blank if not applicable:

- MSc Art
- MSc Biomedical Science
- MSc Cardiology
- MSc Clinical Anatomy
- MSc Dementia
- MSc Neuroscience
- MSc Nursing
- MSc Occupational Therapy
- MSc Physician Associate
- MSc Physiotherapy
- MSc Radiology
- MSc Surgery

1. Please give details about any other postgraduate course not listed above.
2. Is there any other significant teaching the anatomy team undertakes that has not already been captured? If so, please explain.
3. In recent years, what areas has the anatomy department invested in e.g. TEL, embalming?
4. What is your biggest concern for the future of anatomy?
